# Insights into the Antibacterial Activity of Prolactin-Inducible Protein against the Standard and Environmental MDR Bacterial Strains

**DOI:** 10.3390/microorganisms10030597

**Published:** 2022-03-09

**Authors:** Mohd Yousuf, Asghar Ali, Parvez Khan, Farah Anjum, Abdelbaset Mohamed Elasbali, Asimul Islam, Dharmendra Kumar Yadav, Alaa Shafie, Qazi Mohd. Rizwanul Haque, Md. Imtaiyaz Hassan

**Affiliations:** 1 Department of Biosciences, Jamia Millia Islamia, Jamia Nagar, New Delhi 110025, India; yousufbiochem@gmail.com (M.Y.); asg.bstlko@gmail.com (A.A.); qhaque@jmi.ac.in (Q.M.R.H.); 2 Centre for Interdisciplinary Research in Basic Sciences, Jamia Millia Islamia, Jamia Nagar, New Delhi 110025, India; parvezynr@gmail.com (P.K.); aislam@jmi.ac.in (A.I.); 3 Department of Clinical Laboratory Sciences, College of Applied Medical Sciences, Taif University, P.O. Box 11099, Taif 21944, Saudi Arabia; f2016anjum@gmail.com (F.A.); dr.alaa.shafie.tu@gmail.com (A.S.); 4 Department of Clinical Laboratory Science, College of Applied Medical Sciences-Qurayyat, Jouf University, Sakakah 42421, Saudi Arabia; aeelasbali@ju.edu.sa; 5 College of Pharmacy, Gachon University of Medicine and Science, Hambakmoeiro 191, Yeonsu-gu, Incheon City 21924, Korea

**Keywords:** prolactin inducible protein, antibacterial activity, multidrug-resistant bacteria, small proteins

## Abstract

*Background:* Prolactin inducible protein (PIP) is a small secretary glycoprotein present in most biological fluids and contributes to various cellular functions, including cell growth, fertility, antitumor, and antifungal activities. *Objectives:* The present study evaluated the antibacterial activities of recombinant PIP against multiple broad-spectrum MDR bacterial strains. *Methods:* The PIP gene was cloned, expressed and purified using affinity chromatography. Disk diffusion, broth microdilution, and growth kinetic assays were used to determine the antibacterial activities of PIP. *Results*: Disk diffusion assay showed that PIP has a minimum and maximum zone of inhibition against *E. coli* and *P. aeruginosa*, respectively, compared to the reference drug ampicillin. Furthermore, growth kinetics studies also suggested that PIP significantly inhibited the growth of *E. coli* and *P. aeruginosa*. The minimum inhibitory concentration of PIP was 32 µg/mL for *E. coli* (443), a standard bacterial strain, and 64 µg/mL for *Bacillus* sp. (LG1), an environmental multidrug-resistant (MDR) strain. The synergistic studies of PIP with ampicillin showed better efficacies towards selected bacterial strains having MDR properties. *Conclusion:* Our findings suggest that PIP has a broad range of antibacterial activities with important implications in alleviating MDR problems.

## 1. Introduction

Innate immunity plays an important role in human health [1]. During infection, injury, and disease conditions, the metabolism or nutritional homeostasis of the body is disturbed, involving various signaling pathways and secondary messenger molecules [2,3]. Innate immune responses protect our body through various alterations such as modulating membrane composition, antioxidants or cellular redox levels, and cytokines production [4,5]. In addition, the innate immune system provides the first line of defense mechanism against different types of bacteria by secreting multiple types of defense proteins and peptide molecules such as lysozyme, secretary leucoprotease, and lactoferrins that kill or inhibit bacterial growth [6]. The effector molecules are low-molecular-weight peptides and work as multifunctional molecules; one such small protein is a prolactin-inducible protein (PIP), found in different body secretions, where it controls bacterial growth and binds to several other partner molecules to exhibit multiple functions in cancer growth, fertility, and enamel pellicle formation [7,8,9,10,11,12,13].

PIP plays an important function in immune defense and the reproductive system, and its expression is upregulated by prolactin or androgens, whereas it is downregulated by estrogens [14,15]. During pathological condition like corneal disease, keratoconous, dacryliths, and cancer, the expression of PIP get altered in several exocrine tissues or mammary glands such as sweat and salivary glands, and thus acting as a biomarker in such diseases [16,17,18,19]. PIP is a small single polypeptide chain protein expressed in various human body parts, including the salivary gland, lacrimal gland, trachea, prostate, muscle, mammary glands, and lungs. The highest expression of PIP was reported in the salivary gland (~55%) [16,20]. The gene of human PIP has located on chromosome 7q32-36 regions, encoding for a 146 amino-acid-residue-long polypeptide leading to the synthesis of a ~17 kDa protein [16,21,22]. 

PIP is a β-rich glycosylated protein. The crystal structure of PIP demonstrated that β sheets are organized around the hydrophobic amino acid residue and form a sandwich-like structure. PIP contains Asn-X-Ser/Thr motif present at position Asn77-Arg78-Thr79, representing a potential glycosylation site [21]. The structural domain of PIP represents immunoglobulin domains [23,24]. Previous studies on the structure and function of PIP demonstrated its isolation from different biological fluids, where it performs numerous functions with its interacting partner proteins [16,25,26,27]. The presence of immunoglobin domains suggested PIP’s immunological implications supported by multiple studies using in vitro and in vivo (mouse models) presenting the role of PIP in innate and cell-mediated immunity [28,29]. 

The expression of PIP is used as a marker of mammary cell differentiation, and its altered expression is associated with breast cancer progression [29]. Recent studies suggested that the downregulation of PIP promotes osteogenic differentiation of periodontal stem cells [30]. Multiple reports supported the antimicrobial or anti-protozoal roles of PIP. They suggested that reduced levels of antimicrobial or immunomodulatory proteins in tears lead to higher ocular infections and susceptibility to other pathogens such as *Leismania major* [31,32]. PIP acts on oral bacteria (an oral defense mechanism), promoting its clearance from the oral cavity and modulating oral flora [33]. PIP is present in most of the biological/physiological fluids of the human body. However, the antibacterial function of PIP is not well-studied and little work has been conducted on evaluating its antibacterial properties. Thus, we aimed to investigate the antibacterial activity of PIP on multiple microbial strains, including MDR strains. 

## 2. Materials and Methods

### 2.1. Bacterial Strains and Plasmids

The coding region of the *PIP* gene was amplified from total human cDNA through PCR using PIP-specific primers bearing *NdeI* (forward primer) and *XhoI* (reverse primer) restriction sites. The amplified amplicon was digested with *NdeI* and *XhoI, and subsequently* cloned into pET28^a+^ (Novagen, Madison, WI, USA) prokaryotic expression vector. DH5α and BL21 (*DE3*) strains of *E. coli* cells were used for PIP cloning and expression, respectively. Plasmid isolation, restriction enzyme digestion by *NdeI* and *XhoI*, and the ligation experiments were performed as described previously [34]. Luria Broth (DifcoTM, Becton Dickinson, Fisher Scientific, Kansas City, KS, USA) was used for bacterial culture with 50 µg/mL Kanamycin (Sigma, Saint Louis, MO, USA).

### 2.2. Expression and Purification of PIP

The pET28a+-PIP expression construct was transformed in *E. coli* BL21 (*DE3*) following the standard protein expression protocol as described previously [35]. The primary culture grown overnight was used to develop secondary culture containing 50 µg/mL kanamycin and incubated at 37 °C with constant agitation at 180 rpm in an incubator shaker until the absorbance reached 0.6 at 600 nm. The secondary culture was induced by 0.5 M IPTG (Sigma, Saint Louis, MO, USA) and incubated for an additional 4–5 h at 37 °C, 180 rpm.

The cell culture was centrifuged at 7000–8000× *g* for 10 min, and the cell pellet was dissolved in cell lysis buffer having 50 mM Tris–HCl buffer, pH 8.0, 200 mM NaCl, 2% (*v/v*) glycerol, 1 mM β-mercaptoethanol, 0.1 mg/mL lysozyme, 1 mM phenyl methane sulfonyl fluoride (PMSF), and 1% (*v*/*v*) triton X-100 (U. S. Biochemical Corp, Cleveland, OH, USA) and incubated for 1 h at 37 °C. Following the incubation, cell lysate was sonicated on ice for 15 min and centrifuged for 30 min at 13,000 rpm at 4 °C. The supernatant was collected, and protein expression was checked using SDS-PAGE. Ni-NTA affinity chromatography was used for the purification of 6XHis-tag-PIP protein. For this, the clear supernatant was passed through Ni–NTA column pre-equilibrated with Tris buffer (50 mM Tris–HCl, pH 8.0, 200 mM NaCl, 1% *v*/*v* glycerol, 20 mM imidazole). Following protein binding, the column was washed with 50 mL of washing buffer (50 mM Tris–HCl, pH 8.0, 200 mM NaCl, 50 mM imidazole) at 4 °C. Bound protein was eluted with an imidazole gradient (100–300 mM). The eluted fractions were run on the SDS-PAGE to check the purity of the protein. Fractions containing purified protein were pooled and dialyzed in 20 mM Tris–HCl buffer, pH 8.0, 100 mM NaCl, and 2% glycerol. The dialyzed protein was concentrated using Amicon Ultra 5 K device (Merck, Darmstadt, Germany). The concentrated protein sample was further loaded on Hi Trap DEAE-FF (1 mL, 7 mm × 25 mm) column (GE Healthcare, Chicago, IL, USA) pre-equilibrated with 50 mM Tris–HCl buffer, pH 8.0. Bound proteins were eluted with increasing concentration of NaCl (0–1 M) in the 50 mM Tris–HCl buffer, pH 8.0. PIP eluted at 0.50 M NaCl was pooled, concentrated, and stored for further studies. The purity of PIP was tested by SDS-PAGE and validated through Western blot using the luminol method [36].

### 2.3. Antibacterial Activity Assays

Subculturing of standard bacterial strains, including *S. aureus* (MTCC 902), *B. subtilis* (MTCC 736), *P. aeruginosa* (MTCC 2453), *Escherichia coli* (MTCC 443), and environmentally resistant (multidrug-resistant) bacterial isolates, i.e., *Citrobacter werkmanii* (SH 52/MN267555), *E. coli* (SD6/MT577556)*, Bacillus* sp. (LG1/MT576690), and *Citrtobacter* sp. (HK 106/MT576965) was performed on nutrient agar medium through the streaking method [37]. Following the streaking, the plates were kept in the incubator overnight at 37 °C, and the growth of each plate was observed on the next day.

### 2.4. Determination of MIC and MBC 

To determine the MIC of the PIP, the microdilution method was performed according to the standard protocol of NCCL [38]. MIC of the PIP was determined against two Gram-positive (*B. subtilis* and *S. aureus*) and two Gram-negative (*P. aeruginosa* and *E. coli*) bacterial strains. The MIC values were estimated and compared to ampicillin (AMP), used as a control antibiotic. To obtain the stock solution of 10.24 mg/mL for PIP and AMP (standard bacterial drugs), they were dissolved in Tris buffer and sterile water, respectively. The serial dilutions of broth were performed to obtain the final concentrations of 1024, 512, 256, 128, 64, 32, 16, 8, 4, 2, 1, 0.5, 0.25, and 0.125 μg/mL. A control test with buffer was performed following the same dilutions to check whether the buffer or solvent of PIP affected the growth of bacteria. The cultures were incubated at 37 °C for 24 h and compared with blank in terms of turbidity developed by the microbial growth. The minimum concentration of PIP exhibiting no bacterial growth was described as MIC. For the MBC, 10 μL aliquots from each well that showed no growth of microorganism were plated on Mueller–Hinton Agar (MHA) and incubated at 37 °C for 24 h [39,40]. The MIC and MBC values were calculated as per the previously published protocols [41] and compared with the AMP taken as a reference antibiotic. 

### 2.5. Disk Diffusion Assay

Disk diffusion assay was performed to identify the antibacterial efficacy of PIP against standard bacterial strains by following Kirby–Bauer method [42]. Various concentrations of PIP such as MIC/2, MIC and 2MIC were taken on disc for disc diffusion assay [43,44,45,46]. Briefly, the bacterial cells were inoculated in a liquid broth medium and grown overnight at 37 °C. Approximately 10^5^ cells/mL were taken from that liquid broth medium, inoculated into molten nutrient agar medium, and poured into Petri plates [45,47,48,49]. After solidification, autoclaved Whatman papers having 4 mm diameter disks were put at suitable distances over solid agar, and MIC/2, MIC, and 2MIC from the stock solution were put on disks. Plates were incubated at 37 °C overnight, and the next day, ZOI was measured in mm.

### 2.6. Combination Studies of PIP against the Standard Bacterial Strains

The synergistic activity of PIP with standard drug AMP was determined using the microdilution checkerboard method against standard bacterial strains of *E. coli* and *P. aeruginosa* as described previously [50]. AMP were serially diluted in columns from 32,16, 8, 4, 2, 1, 0.5, 0.25, 0.125, and 0.0625 μg/mL, while PIP was diluted in rows from 1024, 512, 256, 128, 64, 32, 16, 8, 4, 2, 1.0, and 0.5 μg/mL, respectively, in a 96-microwell plate to obtain multiple combination. The plates were inoculated with a freshly prepared culture of bacterial isolates and incubated at 37 °C overnight. Following the incubation time, combinatorial MIC was determined as the concentrations at which no visible growth occurred. Using the formula given below, the synergy of compounds in terms of FICI (fractional inhibitory concentration index) was calculated [41]:

ΣFIC = FICA + FICB = (CA/MICA) + (CB/MICB)

where MICA and MICB are the MICs of drugs A and B alone, respectively, and CA and CB are the concentrations of the drugs in combination, respectively, in all of the wells corresponding to an MIC (isoeffective combinations).

Synergy and antagonism were defined by FICI indices ≤ 0.5 and >4, respectively, and ‘indifferent’ was defined by 1 < FICI ≤4.

### 2.7. Growth Kinetics Assay

Based on previous experiments, we have selected *E. coli* and *P. aeruginosa* for these experiments. Bacterial cells were freshly revived by sub-culturing on the Luria agar plate. To obtain the fresh cultures for the experiments, an inoculum was transferred into the Luria broth and was grown overnight at 37 °C. Different concentrations of PIP, equivalent to MIC/2, MIC, and 2MIC, were added separately to the respective conical flasks containing inoculated medium and incubated at 37 °C. Positive control was also taken to observe the full growth. To observe the growth kinetics of the cultures, 1 mL aliquot of each sample was taken out from the culture flask and growth was measured at 590 nm turbidometrically (optical density) using Thermo Multiskan spectrophotometer after each one-hour interval [51]. To determine the effect of PIP on bacterial growth, a graph was plotted between OD versus time duration (hours) to obtain a growth curve and further analysis [52]. 

### 2.8. Effect of PIP on Environmental Resistant Bacterial Strains

Antibacterial activity of PIP was investigated against a variety of environmental resistant strains such as HK 106 (*Citrobacter* sp.), LG 1 (*Bacillus* sp.), SD 6 (*Escherichia coli*), and SH52 (*Citrobacter werkmanii*). All bacterial strains were isolated from different environmental (Lake, Pond, effluent from slaughterhouse and wastewater treatment plant) water samples. The standard broth dilution method was used to determine the MIC of these isolates and compare the MIC values to the conventional antibiotics such as ampicillin (a penicillin analog). The microdilution checkerboard method has been used to examine the synergistic activity of the PIP with the standard antibiotic AMP against *Escherichia coli* and *Citrtobacter werkmanii*. The procedure above was used for the MIC and synergistic activity [41,53,54,55]. 

## 3. Result and Discussion

### 3.1. Cloning, Expression, and Purification of PIP 

**Figure 1:**
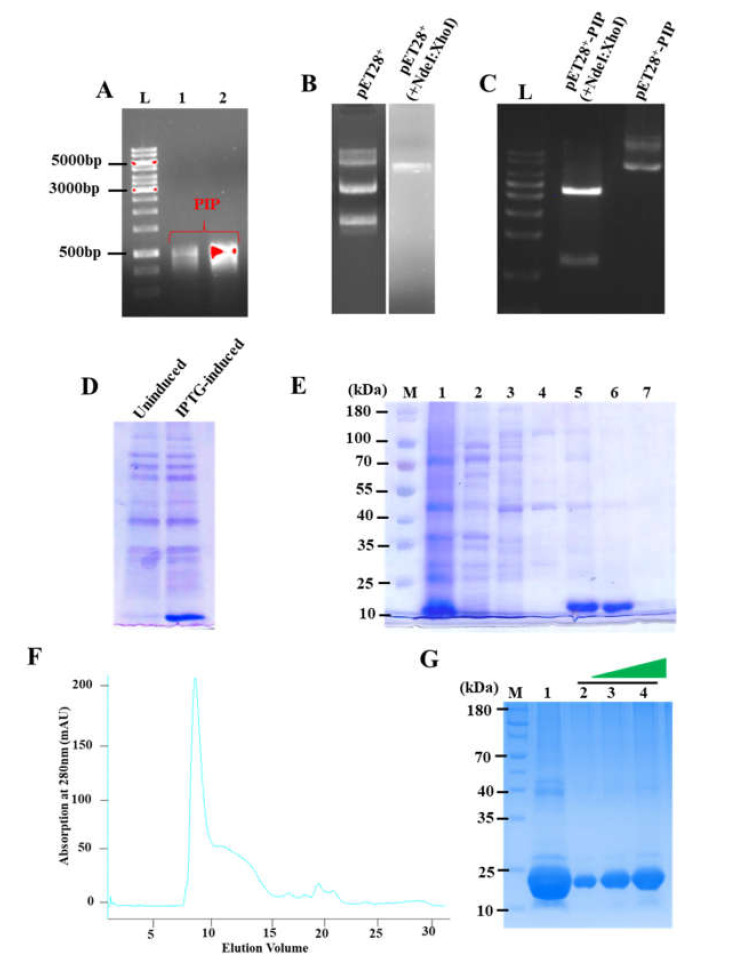
Cloning, expression, and purification of PIP: (**A**) Amplification of PIP gene through PCR. Lane 1: Marker; Lane 2 and 3 are amplified products of the PIP gene. (**B**) Lane 1: purified pET28^a+^ plasmid, Lane 2: pET28^a+^ plasmid digested with *NcoI* and *XhoI.* (**C**) Restriction digestion studies of PIP expression clone, Lane 1: marker, Lane2: digested pET28^a+^-PIP construct with *NcoI* and *XhoI*, Lane 3: undigested pET28^a+^-PIP construct. (**D**) Expression of recombinant PIP using *E. coli* as an expression host showing un-induced and IPTG-induced bacterial culture having PIP construct. (**E**) Elution profile of recombinant PIP protein: Lane 1: Marker, Lane 2: supernatant from whole cell lysate before binding to Ni-NTA column; Lane 3: supernatant after Ni-NTA binding; Lane 4: elution from 20 mM imidazole elution buffer; Lane 5: elution from 50 mM imidazole buffer; Lane 6: elution from 200 mM imidazole buffer; Lane 7: elution from 300 mM imidazole buffer; Lane 8: elution from 500 mM imidazole buffer. (**F**) PIP elution profile from ion-exchange chromatography. (**G**) The 15% SDS-PAGE for PIP elutions; Lane 1: Marker, Lane 2: impure PIP before loading to ion-exchange chromatography, Lane 3–5: showing increasing concentration gradients (5 µg, 10 µg and 15 µg, respectively) of purified PIP eluted from ion-exchange chromatography.

The *PIP* gene (from plasmid pcDNA) was amplified using a gene-specific PCR, with *Ndel* and *XhoI* sites in the forward and reverse primers, respectively. The size of the amplified product was 366-bp (Figure 1A). The amplified gene product having selected restriction sites was digested with *NcoI* and *XhoI* and subsequently ligated into the pET28a+ backbone digested with similar restriction enzymes (Figure 1B). The ligated product was transformed into *E. coli* and positive colonies were selected and confirmed using colony PCR. The positive clones were further confirmed using restriction digestion through *NcoI* and *XhoI* endonucleases (Figure 1C). Finally, the constructed plasmid was verified by DNA sequencing (Figure S1).

The confirmed plasmid construct pET28a+ with PIP was transformed into BL21 (*DE3*) strain of *E. coli* for protein expression. The recombinant protein expression was performed at 37 °C by inducing the bacterial cultures with 0.5 mM IPTG for 4–5 h. The over-expression of PIP was confirmed through SDS-PAGE, showing a prominent protein band corresponding to PIP with an apparent molecular mass of ~14 kDa (Figure 1D). The purification of PIP follows two-step processes. In the first step, PIP was purified using Ni-NTA chromatography (Figure 1E), followed by the second step of purification using ion-exchange chromatography (Figure 1F,G). A single band corresponding to the size of PIP was observed on the SDS-PAGE, confirming the purity of PIP (Figure 1G).

### 3.2. Determination of MIC and MBC

Multiple studies reported the presence of PIP in several body fluids, showing antibacterial activities [7,56,57]. To study the antibacterial activities of PIP, in particular, we have successfully cloned, expressed, and purified the PIP using a prokaryotic expression system. It is important to mention that the recombinant protein isolated from the prokaryotic expression system is devoid of post-translational modifications. We previously reported the crystal structure of PIP isolated from seminal human plasma. We noticed comparable structural properties of recombinant PIP, thus suggesting that recombinant PIP might be as active as the protein purified from body fluids [22,23]. 

Initially, the antibacterial activity of PIP was evaluated through the zone of inhibition (ZOI) measurements in diameter (mm) on MHA plates and serial dilution of broth to observe the growth of bacteria by measuring the turbidity of cultures. The estimated MIC value for the PIP against *B. Subtilis* and *P. aeruginosa* was 64 µg/mL, wh. In contrast, for *E. coli* and *S. aureus* the MIC values were 32 µg/mL and 128 µg/mL, respectively (Table 1). AMP was used as a reference antibiotic (Positive control). The results suggested that PIP has the highest inhibitory effect against *E. coli* among the tested strains. However, the MIC value for *S. aureus* bacterial strain appears to be comparatively high. Interestingly, PIP showed a similar profile activity (MIC values) towards *B. subtilis* and *P. aeruginosa* strains. 

In addition, the MBC values of PIP range from 128 to 512 µg/mL (Table 1). The ratio of MBC to MIC is used to characterize the antibacterial activity of any compound. If the MBC/MIC ratio was ≤2, the compounds were termed bactericidal, and they were termed bacteriostatic if the ratio was between 2 and 16 [43,45,58,59,60,61]. The results shown in Table 1 suggested that the PIP showed bactericidal and bacteriostatic effects based on the tested bacterial strains.

### 3.3. Disk Diffusion Assay

**Figure 2:**
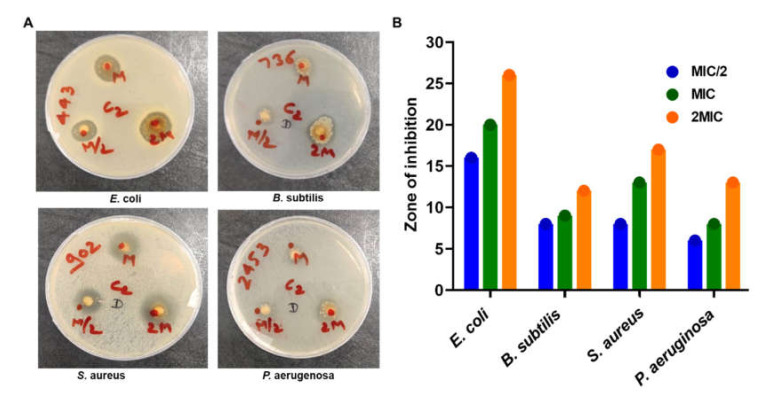
Zone of inhibition assay. (**A**) Representative images of culture plates showing PIP mediated growth inhibition of *Bacillus subtilis*, *E. coli, Staphylococcus aureus*, and *Pseudomonas aeruginosa*. (**B**) Quantification of ZOI studies for *Bacillus subtilis*, *E. coli, Staphylococcus aureus*, and *Pseudomonas aeruginosa*.

**Figure 3:**
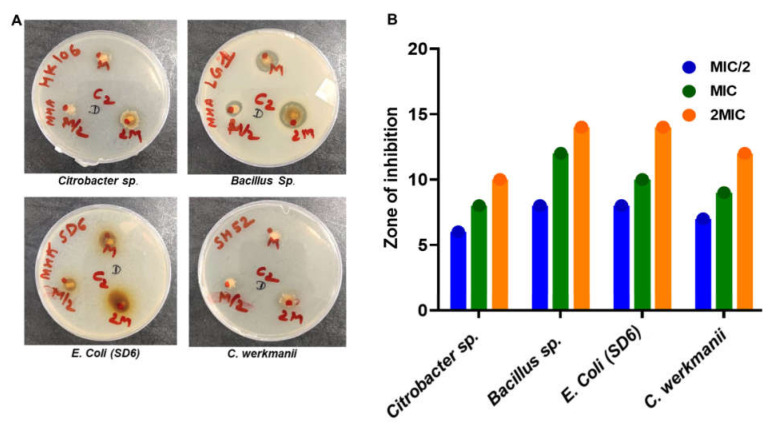
Zone of inhibition assay: (**A**) Representative images of culture plates showing PIP mediated growth inhibition of *Citrobacter* sp., *Bacillus* sp., *E. Coli (SD6)*, and *C. werkmanii*; (**B**) Quantification of ZOI studies for *Citrobacter* sp., *Bacillus* sp., *E. coli (SD6)*, and *C. werkmanii*.

The disc diffusion test is a widely accepted and primary test to see the susceptibility of bacteria or bacterial isolates towards antibiotics or any antibacterial molecules [46,49,62,63]. To further evaluate the antibacterial activity of PIP, following the serial dilution method, the disk diffusion assay was used. The disk diffusion experiment results revealed that PIP showed the lowest and highest ZOI against *P. aeruginosa* and *E. coli*, respectively (Figure 2). In Gram-negative *E. coli* culture, the treatment with PIP leads to a clear ZOI of 16, 20, and 26 mm around the disc of MIC/2, MIC, and 2MIC, respectively. While in the case of *P. aeruginosa* (2453), 6, 8, and 13 mm clear ZOI 2343 measured around the disks of the MIC/2, MIC, and 2MIC concentrations, respectively (Figure 2, Table 2). Around the disks of MIC/2, MIC, and 2MIC, the observed ZOI was 8, 9, and 12mm with *B. subtilis* and 8, 13, and 17 with *S aureus,* respectively. Antibacterial activities of PIP using agar diffusion assay are presented in Figure 2 and Figure 3. Diffusion assay results in terms of ZOI diameter (mm) for standard bacterial strains are given in Table 2.

### 3.4. Synergistic Antibacterial Activity of PIP 

The development of multidrug resistance (MDR) in bacteria is a leading cause of bacterial-infection-related deaths [64,65,66,67,68,69]. Therefore, the development of combinatorial therapeutic strategies or synergistic approaches offered an effective therapeutic regimen to deal with MDR problems [64,70,71,72,73]. To investigate the synergistic effect of PIP towards the antibacterial activity of the standard drug (ampicillin), PIP was further evaluated in combination with AMP against selected standard bacterial strains. Similar synergistic experiments were also performed with environment MDR bacterial strains. Interestingly, the results of synergistic studies showed that PIP with AMP significantly enhanced the antibacterial activity of standard drugs. The results of synergistic studies are summarized in Table 3.

The results showed a substantial increment in the antibacterial activity of PIP against *E. coli* and *P. aeruginosa* strains when used in combination with AMP. The resistance pattern of all these bacterial isolates toward various antibiotics is described in Table 4. The outcomes of synergistic studies suggested the potential implications of PIP towards the development of future combinatorial therapeutic strategies to alleviate antimicrobial resistance. 

### 3.5. Growth Kinetics

**Figure 4:**
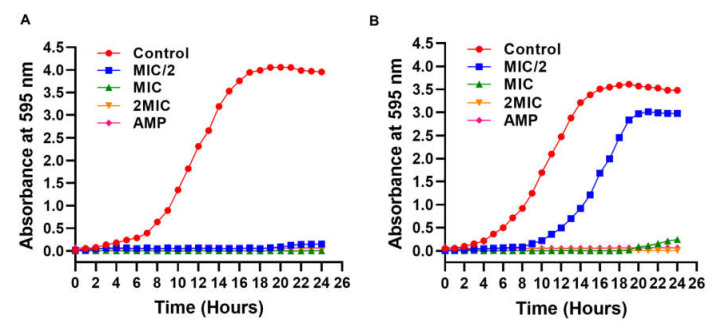
Growth kinetics study of (**A**) *E. coli*, (**B**) *P. aeruginosa* at different MIC concentrations of PIP. Ampicillin (AMP) was taken as a positive control.

The kinetic growth studies provide an important tool to see the influence of substrate concentration or a molecule towards the specific growth rate (versus time) and provide a method to evaluate drug susceptibility to microbial growth [74,75]. Therefore, growth kinetics studies were performed to determine the effect of PIP on the growth of *E. coli* and *P. aeruginosa*. For this, we take untreated cultures as a negative control, and AMP-treated culture was taken as a positive control. The results showed that the growth curve of untreated bacterial cells (control) follows the standard growth curve with a clear lag, exponential or log, brief stationery, and decline phases of the bacterial cell. On the other hand, at 2MIC, MIC, and MIC/2 concentration values of PIP, no growth was observed until 20 h of incubation in *E. coli* growth experiments (Figure 4). In the case of *P. aeruginosa* at 2MICconcentration values of PIP, no growth was observed until 24 h. At MIC concentration values, a slight increase in the culture growth was observed after 20 h.

In contrast, at the MIC/2 concentration, the lag phase was increased from 3 to 8 h, the log phase was significantly increased from 9 h to 19 h, and following the log phase, a short stationary phase was observed from 20 h to 22 h, then a decline phase was observed. Moreover, PIP completely inhibited the growth of both bacterial strains at MIC and 2MIC concentrations. No growth was observed in AMP-treated bacterial cells for 24 h in both selected bacterial strains.

### 3.6. Effect of PIP on Environmental MDR Strains

The MIC value for the PIP against environmental MDR isolates was observed from 64–512 µg/mL. Minimum MIC values 64 µg/mL for *LG1 64,* whereas maximum MIC values 512 µg/ml for SD6 were observed among tested MDR isolates (as shown in Table 1). Environmental MDR isolates SH52, LG1, SD6 showed a significant decrease as 4, 3, 2-fold MIC values compared to AMP, respectively, while HK106 showed nearly the same MIC value for PIP and AMP (Table 1). MBC values ranges for the resistant strain varies from > 1024 to 512 µg/mL. If the MBC/MIC ratio was 2 to 8, and the compounds were termed bactericidal and bacteriostatic. The PIP has various bactericidal and bacteriostatic effects on environment-resistant bacterial isolates. The zone of inhibition assays for PIP against environmental-resistant bacterial isolate *LG1* shows a maximum ZOI of 8mm at MIC/2 concentration and of 12 mm at MIC concentration. Moreover, at 2MIC concentration, the maximum ZOI was 14 mm. At the same time, *Citrobacter werkmanii* (SH 52) shows a ZOI of 7 mm at the MIC/2 concentration, 9 mm at the MIC concentration, and 12 mm at the 2MIC concentration. Furthermore, the combinational (PIP and AMP) studies against SD6 and SH52 showed that the PIP has a differential mode of interaction (FICI = 1.5) with AMP against SD6. In contrast, it showed the synergistic mode of interaction (FICI = 0.0097) against SH52 (Table 3).

## 4. Conclusions

To study the antibacterial potential of PIP, we cloned, expressed, and purified PIP using a prokaryotic expression system. The antibacterial activities of PIP were evaluated against a broad spectrum of bacterial strains. The outcomes of the present study suggest that PIP significantly inhibited a wide range of bacterial strains, including standard and environmental MDR strains. The enhanced antibacterial potential of standard antibiotics such as ampicillin in synergistic studies of PIP makes it an important investigatory molecule towards developing combinatorial antibacterial approaches to deal with the rising burden of antimicrobial resistance. This study indicates PIP’s future investigations as a novel bioactive molecule for developing antibacterial therapies and as a preservative agent in the food, cosmetic, and medicine industries.

## Figures and Tables

**Table 1:** MIC and MBC Values (µg/mL) of PIP and AMP against bacterial strains.

	Isolates			MIC	MBC	MBC/MIC
PIP (µg/mL)	Standard Strains	443	*E. coli*	32	128	4
		736	*B. subtilis*	64	256	4
		902	*S. aureus*	128	512	4
		2453	*P. aeruginosa*	64	128	2
	Environmental MDR strains	HK 106	*Citrobacter* sp.	128	>1024	8
		LG 1	*Bacillus* sp.	64	512	8
		SD 6	*E.coli*	512	1024	2
		SH 52	*B. werkmanii*	128	512	4
Amp (µg/mL)	Standard Strains	443	*E. coli*	1	-	-
		736	*B. subtilis*	0.5	-	-
		902	*S. aureus*	0.25	-	-
		2453	*P. aeruginosa*	4	-	-
	MDR strains	HK 106	*Citrobacter* sp.	128	-	-
		LG 1	*Bacillus* sp.	256	-	-
		SD 6	*E.coli*	1024	-	-
		SH 52	*B. werkmanii*	1024	-	-

Minimal bactericidal concentration values for the PIP against eight studied bacterial isolates. *Citrobacter* sp. (HK106/MT576965) shows maximum MBC value (>1024 µg/mL), while *Pseudomonas aeruginosa* (2453) and *E. coli* (443) shows minimal MBC value (128 µg/mL) among the tested isolates.

**Table 2:** Zone of inhibition (in mm) as measured around the disk of various concentrations of compound.

Types of Bacterial Isolates	Isolates Code	Bacterial Strain	Zone of Inhibition at Different Concentrations of Test Compound		
			MIC/2	MIC	2MIC
Standard Bacterial Strains	443	*E. coli*	16	20	26
	736	*B. subtilis*	8	9	12
	902	*S. aureus*	8	13	17
	2453	*P. aeruginosa*	6	8	13
Environmental MDR Bacterial strains	HK 106	*Citrtobacter* sp.	6	8	10
	LG 1	*Bacillus* sp.	8	12	14
	SD 6	*E. coli*	8	10	14
	SH 52	*C. werkmanii*	7	9	12

**Table 3:** Synergistic antibacterial activity of PIP with AMP.

Bacterial Strain	MIC Alone (μg/mL)		MIC in Combination (μg/mL)		FICI *	Mode of Interaction
	PIP	AMP	PIP	AMP		
*E.coli* (443)	32	1	2	0.125	0.187	Synergistic
*P. aeruginosa *(2453)	128	4	8	1	0.312	Synergistic
*E.coli* (SD 6)	512	1024	512	512	1.5	Indifferent
*Citrobacter werkmanii* (SH 52)	128	1024	1	2	0.0097	Synergistic

A FICI of ≤ 0.5 was defined as synergy, a FICI of > 0.5 but ≤ 4.0 was defined as no interaction, and a FICI of > 4.0 was defined as antagonism. * FICI: fractional inhibitory concentration index.

**Table 4:** Resistance pattern of environmental isolates.

Isolates	Strain	Accession No	Resistance Pattern	MIC (µg/mL) for AMP	Site
HK 106	*Citrtobacter* sp.	MT576965	AMP, ETP, IPM, CX, P/T CZ, PB, AK, CIP, CL, TR	128	Hauz Khas lake
LG 1	*Bacillus* sp.	MT576690	AMP, CX, A/S, RIF, CZ, TR	256	Lodhi Garden Pond
SD 6	*Escherichia coli*	MT577556	AMP, IMP, CX, RIF, CZ, PB, AK, TE, CIP, LE	1024	Shaheen Bagh Drain
SH 52	*Citrtobacter werkmanii*	MN267555	ETP, CX, RIF, CZ, PB, CL, TR	1024	Ghazipur Slaughterhouse effluent

AMP: Ampicillin, A/S: Ampicillin/Sulbactam, CX: Cefoxitin, IPM: Imipenem, P/T: Piperacillin/Tazobactam, CZ: Cefazolin, ETP: Ertapenem, RIF: Rifampicin AK: Amikacin, CL: Colistin, PB: Polymyxin B, TE: Tetracycline, CIP: Ciprofloxacin, LE: Levofloxacin, TR: Trimethoprim.

## Data Availability

Not applicable.

## Supplementary Materials

The following supporting information can be downloaded at: https://www.mdpi.com/article/10.3390/microorganisms10030597/s1. Figure S1: Sequence of PIP gene.
