# Supplementary material for: Insights into the Antibacterial Activity of Prolactin-Inducible Protein against the Standard and Environmental MDR Bacterial Strains
Source: Microorganisms. 2022 Mar 9;10(3):597. doi: 10.3390/microorganisms10030597 (PMC8950685; doi:10.3390/microorganisms10030597)
Supplement: Supplementary file 1 [file microorganisms-10-00597-s001.zip › microorganisms-1634335-supplementary.pdf]

>H30195-U9324CI260-2-T7ter
TTCGGGCTTTGTTAGCAGCCGGATCTCAGTGGTGGTGGTGGTGGTGCTCGAGTTATTCTACCTTTAGGATT
TCAATAGTATAAAACCGGTTGTTTTTGATGGGGATTACAGCAGCATCATCAGGGCAGATGCCTAATTCCCG
AATAACATCAACGACGGCTGCAATTTGCACAGTTCTGTTGGTGTAAAAGTCCCAGTAGAAGGTTTTTGGAT
TGTCGTCACATAGGCAGGCAGTATACTTATAGTTAAATGCACCTTGTAGAGGGATGCTGCTAATGAGGTAA
GTTTTAACCACCATGCATTCTTTCAATTCTGTTTGAACTGCAAGCACTGCAGTGACTTCGTCATTTGGACG
TACTGACTTGGGAATGTCAAAATTCTTTATTATGATCTTCCGAGTGTTGTCCATATGGCTGCCGCGCGGCAC
CAGGCCGCTGCTGTGATGATGATGATGATGGCTGCTGCCCATGGTATATCTCCTTCTTAAAGTTAAACAAA
ATTATTTCTAGAGGGGAATTGTTATCCGCTCACAATTCCCCTATAGTGAGTCGTATTAATTTCGCGGATCG
AGATCTCGATCCTCTACGCCGGACGCATCGTGGCCGGCATCACCGGCGCCACAGGTGCGGTTGCTGGCGC
CTATATCGCCGACATCACCGATGGGGAAGATCGGGCTCGCCACTTCGGGCTCATGAGCGCTTGTTTCGGCG
TGGGTATGGTGGCAGGCCCCGTGGCCGGGGGACTGTTGGGCGCCATCTCCTTGCATGCACCATTCCTTGC
GGCG

Fig.S1: Sequence of PIP gene
